# Molecular Insights into Human Transmembrane Protease Serine-2 (TMPS2) Inhibitors against SARS-CoV2: Homology Modelling, Molecular Dynamics, and Docking Studies

**DOI:** 10.3390/molecules25215007

**Published:** 2020-10-29

**Authors:** Safaa M. Kishk, Rania M. Kishk, Asmaa S. A. Yassen, Mohamed S. Nafie, Nader A. Nemr, Gamal ElMasry, Salim Al-Rejaie, Claire Simons

**Affiliations:** 1 Pharmaceutical Medicinal Chemistry Department, Faculty of Pharmacy, Suez Canal University, Ismailia 41522, Egypt; 2 Microbiology and Immunology Department, Faculty of Medicine, Suez Canal University, Ismailia 41522, Egypt; raniakishk@med.suez.edu.eg; 3 Pharmaceutical Organic Chemistry Department, Faculty of Pharmacy, Suez Canal University, Ismailia 41522, Egypt; asmaa_yaseeen@pharm.suez.edu.eg; 4 Chemistry Department, Faculty of Science, Suez Canal University, Ismailia 41522, Egypt; Mohamed_nafie@science.suez.edu.eg; 5 Endemic and Infectious Diseases Department, Faculty of Medicine, Suez Canal University, Ismailia 41522, Egypt; nadernemr@med.suez.edu.eg; 6 Faculty of Agriculture, Suez Canal University, Ismailia 41522, Egypt; gamal.elmasry@agr.suez.edu.eg; 7 Department of Pharmacology & Toxicology, College of Pharmacy, King Saud University, Riyadh 11564, Saudi Arabia; rejaie@ksu.edu.sa; 8 School of Pharmacy & Pharmaceutical Sciences, Cardiff University, King Edward VII Avenue, Cardiff CF103NB, UK; simonsc@cardiff.ac.uk

**Keywords:** TMPS2, SARS-CoV2, homology model, molecular dynamics

## Abstract

The severe acute respiratory syndrome coronavirus 2 (SARS-CoV2), which caused novel corona virus disease-2019 (COVID-19) pandemic, necessitated a global demand for studies related to genes and enzymes of SARS-CoV2. SARS-CoV2 infection depends on the host cell Angiotensin-Converting Enzyme-2 (ACE2) and Transmembrane Serine Protease-2 (TMPRSS2), where the virus uses ACE2 for entry and TMPRSS2 for S protein priming. The TMPRSS2 gene encodes a Transmembrane Protease Serine-2 protein (TMPS2) that belongs to the serine protease family. There is no crystal structure available for TMPS2, therefore, a homology model was required to establish a putative 3D structure for the enzyme. A homology model was constructed using SWISS-MODEL and evaluations were performed through Ramachandran plots, Verify 3D and Protein Statistical Analysis (ProSA). Molecular dynamics simulations were employed to investigate the stability of the constructed model. Docking of TMPS2 inhibitors, camostat, nafamostat, gabexate, and sivelestat, using Molecular Operating Environment (MOE) software, into the constructed model was performed and the protein-ligand complexes were subjected to MD simulations and computational binding affinity calculations. These in silico studies determined the tertiary structure of TMPS2 amino acid sequence and predicted how ligands bind to the model, which is important for drug development for the prevention and treatment of COVID-19.

## 1. Introduction

Emerging respiratory diseases such as severe acute respiratory syndrome coronavirus (SARS) is a vital threat to public health. New emerging diseases remain a cause of outbreaks, epidemics, and pandemics [1,2]. A new fatal respiratory virus in Wuhan, China, was recently identified in mid-December 2019. The RNA virus, isolated from the patients’ bronchoalveolar fluid, was investigated by Metagenomic RNA Sequencing. This RNA virus was identified by the phylogenetic study of the complete viral genome to be a member of the Coronaviridae family; the SARS-like coronavirus community (Betacoronavirus genus, Sarbecovirus subgenus) [3,4,5]. The new emerging respiratory virus was named Severe Acute Respiratory Syndrome Corona Virus 2 (SARS-CoV2) by the World Health Organization (WHO) and the disease is now referred to as coronavirus-19 (COVID-19).

Coronaviruses are species of Nidoviral viruses, including Coronaviridae, Arteriviridae, Roniviridae, and Mesoniviridae [6,7]. The Coronaviridae virus family is divided into two subfamilies: four genera coronavirinae (Alpha, Beta, Gamma, and Delta Coronavirus), and torovirinae [8]. The genera of the Beta coronavirus include respiratory viruses such as SARS, MERS coV, and SARS-CoV2.

**Figure 1:**
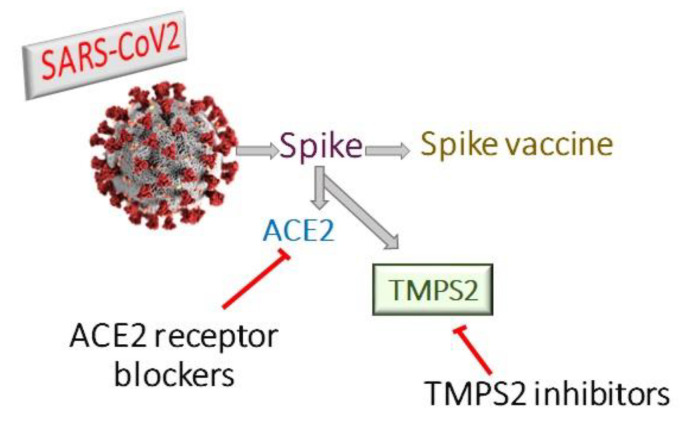
Therapeutic approaches for treatment of covid-19 either by ACE2 receptor blockers, TMPS2 inhibitors or spike vaccine.

Coronaviruses encode four structural proteins in their genomes: Spike (S), Glycoprotein (E), Nucleocapsid (N), and Envelope (E) proteins. The viral attachment protein, which may bind the virion to a host receptor, is considered to be the (S) protein [8]. Angiotensin Converting Enzyme 2 (ACE2) is part of the renin-angiotensin system and its expression protects against acute respiratory distress [9,10]. SARS-CoV employs ACE2 as a host cell entry receptor [11,12]. ACE2, with some mutations in key residues of the SARS-CoV2 (S) protein, was also recognized as the entry receptor for SARS-CoV2 [13,14]. There are, however, indications that the SARS-CoV2 (S) protein binds to the receptor with an affinity much higher than that observed for the SARS-CoV (S) protein, which may play a major role in the rapid spread of COVID-19 worldwide [15]. ACE2 had been considered as the entry receptor [11,16], while (S) protein priming is performed by the transmembrane serin proteases type II TMPRSS2 [17,18,19]. However, the viral spread and pathogenesis of the infected host was shown to involve TMPRSS2 activities [20,21,22] (Figure 1). The TMPRSS2 gene codes for the TMPS2 protein, which is present in all possible targets of SARS-CoV-2 infection, including airway epithelial cells, cardiac endothelium, microvascular endothelial cells, kidney, and digestive tract [23]. Therefore, targeting TMPS2 might represent a suitable approach for both preventing viral infection and treatment of severely ill patients [24].

The TMPS2 inhibitors; camostat mesylate and nafamostat mesylate were found to slow down cell infection by SARS-CoV in preclinical models [25,26,27]. Nafamostat and camostat have anti-inflammatory activity in the airways by reducing inflammatory cytokine production in a chronic allergen-induced asthma model [28,29]. Furthermore, nafamostat can inhibit the coagulation and fibrinolytic systems, the kallikreinkinin system and activate protease-activated receptors [30].

Consequently, the anti-inflammatory, anti-coagulant, and fibrinolytic properties of camostat and nafamostat might contribute to the reduction of symptoms and complications occurring in COVID-19 patients. Both agents are approved in Japan for treatment of pancreatitis [31] and nafamostat is also used as an anticoagulant in extracorporeal circulation [32]. The introduction of nafamostat in addition to antivirals such as lopinavir and hydroxychloroquine in the treatment of elderly COVID-19 patients with pneumonia, showed clinical and radiological improvement without significant adverse effects [33].

Gabexate and sivelestat are serine protease inhibitors marketed in Italy and Japan for the treatment of pancreatitis and have a mechanism similar to nafamostat and camostat (i.e., inhibition of TMPS2).

To our knowledge, there is no crystal structure for TMPS2 available in the Protein Data Bank (PDB), which necessitates the construction of a three-dimensional (3D) structure model to determine the protein architecture and binding interactions of TMPS2 inhibitors to facilitate the improvement of drug design research against COVID-19.

## 2. Results and Discussion

### 2.1. Homology Searching and Template Identification

**Figure 2:**
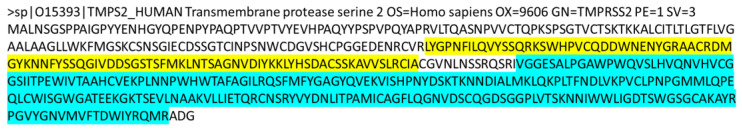
The amino acid sequence of TMPS2 in FASTA format showing the scavenger receptor cysteine-rich (SRCR) domain highlighted in yellow (amino acid sequence 150–242) and the serine peptidase domain highlighted in cyan (amino acid sequence 265–489).

The ExPASy proteomics server was used to obtain the amino acid sequence of the target protein TMPS2. Using the UniProtKB database from the ExPASy server, the amino acid sequence of TMPS2 Homo sapiens in FASTA format was obtained (Figure 2). This protein has a UniProt identifier O15393-1 [34] and is composed of 492 amino acid residues.

Initial screening for suitable templates was carried out by BLAST analyses against the protein data bank (PDB) using both SWISS-MODEL [35] and NCBI blastp [36] (Table 1). SWISS-MODEL analysis revealed 5CE1 and 1Z8G as optimal templates, both of which are crystal structures of transmembrane protease serine 1 with 33.61 and 33.52% identity, respectively. NCBI blastp analysis identified 6KD5_B, transmembrane protease serine 13 (peptidase domain) as the first hit with 45.19% identity, followed by a group of human plasma kallikrein crystal structures (e.g., 6O1G_A) with ~42% identity.

### 2.2. Homology Modelling

Homology models for TMPS2 were generated using SWISS-MODEL from the four identified templates, which were subsequently evaluated using MolProbability [37], ProSA [38] and Verify3D [39] to assess the quality of the models, the data of which is provided in Table 2.

**Figure 3:**
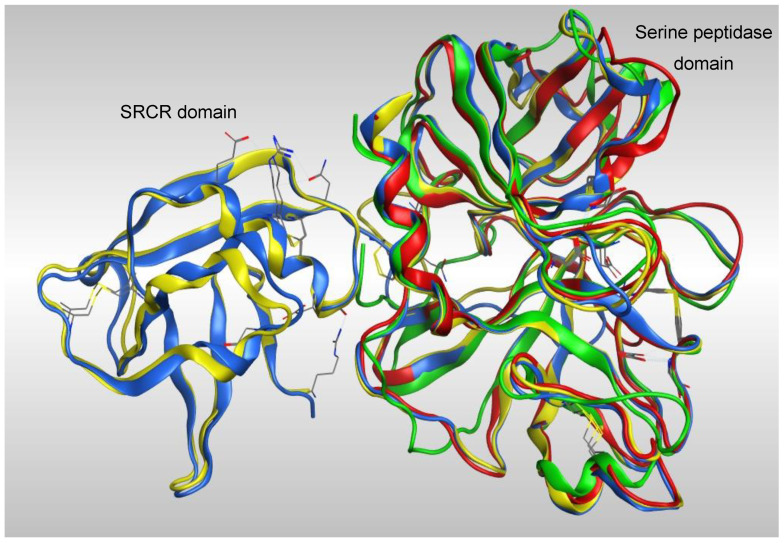
Overlap of the four TMPS2 models generated from crystal structures 5CE1_A (blue), Please ensure that intended meaning has been retained. 1Z8G_A (yellow), 6KD5_B (red) and 6O1G_A (green).

Overlap of the four models (Figure 3) showed a high degree of homology between all four models in the serine peptidase domain, however the greatest coverage was with the models generated from TMPS1 crystal structures 5CE1 and 1Z8G, which have both the scavenger receptor cysteine rich (SRCR) and serine peptidase domains.

**Figure 4:**
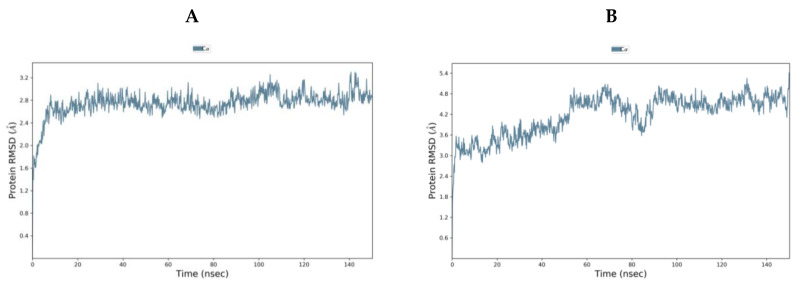
RMSD [Å] plot with respect to time in nanoseconds during 150 ns MD stimulation of (**A**) TMPS2 model 1 (5CE1_A template) and (**B**) TMPS2 model 2 (1Z8G_A template).

TMPS2 models 1 (5CE1_A) and 2 (1Z8G_A) were comparable using Ramachandran plot, Verify3D and ProSA (Tables S1 and S2 and Figures S1–S6 in Supplementary Materials). To evaluate further, both models were subjected to 150 ns molecular dynamics (MD) simulations using the Desmond programme of Maestro [40]. Model 1 started from the Root Mean Square Deviation (RMSD) 1.40 Å and rapidly reached an equilibrium plateau with a final RMSD of 2.86 Å at 150 ns. Model 2 started with a higher RMSD of 2.10 Å but showed more fluctuation and only began to plateau at around 90 ns (RMSD 4.63 Å) with a final RMSD of 4.81 Å at 150 ns (Figure 4). Overlap of the proteins after MD simulation diverged in the SRCR domain but showed better alignment in the serine peptidase domain (Figure S7 in Supplementary Materials). From MD simulations, model 1 would appear to be the more stable system and so was chosen for further studies.

### 2.3. TMPS2 Structure Evaluation

A blastp analysis was performed using the NCBI server, query transmembrane protease serine against human sequences in the swissprot database [41]. Details for TMPS2 (O1593) and the closest five matches were obtained from the Uniprot server and are presented in Table 3.

**Figure 5:**
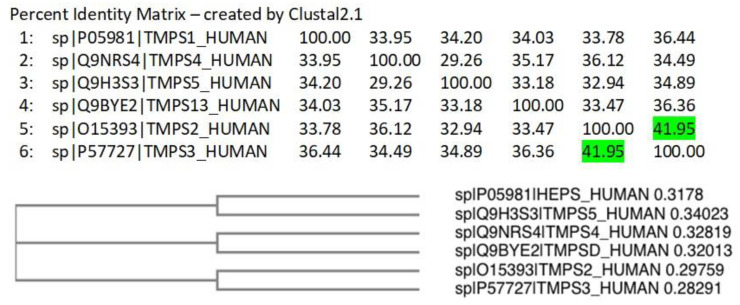
Percent identity matrix and phylogenic cladogram generated from the Clustal alignment of TMPS proteins.

A Clustal Omega [42] alignment was performed on the TMPS proteins. The three amino acids (His, Asp, Ser) that form the catalytic triad required for peptide bond hydrolysis, the cleavage site (Arg-Ile/Val-Val-Gly-Gly/Ile) responsible for proteolytic activation, and the disulphide forming cysteine residues are highly conserved (Figure S8 in Supplementary Materials). TMPS2 most closely aligns with TMPS3 (41.95%) as illustrated from the percent identity matrix and the cladogram (Figure 5).

**Figure 6:**
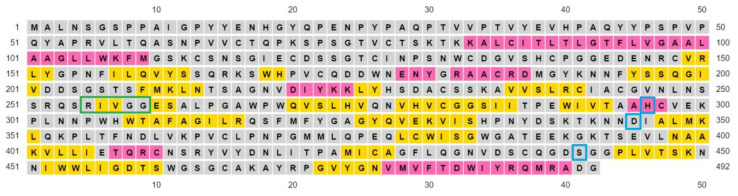
Amino acid sequence of TMPS2 showing α-helices coloured in pink, β-sheets coloured in yellow, loops coloured in grey, the catalytic triad residues are surrounded in cyan and the cleavage site is surrounded in green.

**Figure 7:**
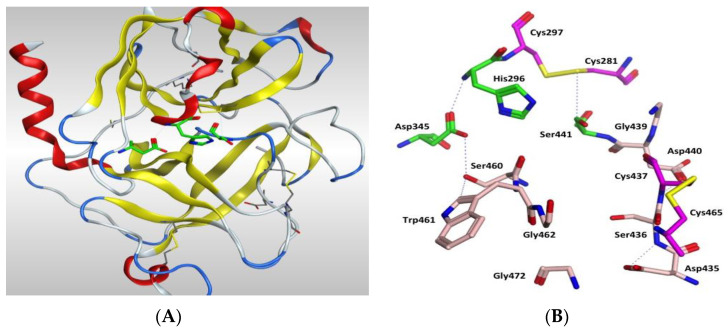
(**A**) 3D image of TMPS2 with the position of the catalytic triad (green) in the serine peptidase domain shown. The two six-stranded β-barrels (yellow) are positioned either side of the catalytic triad. (**B**) Serine peptidase active site showing the catalytic triad (green), the amino acids that form the S1 pocket (light pink) and the two disulfides Cys281-Cys297 and Cys437-Cys465 (magenta).

The protein structure is stabilised by five disulphide bonds, which are found in the SRCR non-catalytic domain [43], formed by Cys172–Cys231 and Cys185–Cys241, and in the serine peptidase domain by Cys281–Cys297, Cys410–Cys426 and Cys437–Cys465. The catalytic triad of His296, Asp345 and Ser441 spans the active site with Ser441 on one side and Asp345 and His296 on the other side of the S1 pocket (Figure 6 and Figure 7). Asp435 sits at the base of the S1 pocket and Ser436 and Asp440 form the right wall. Asp435, Gly462 and Gly472 create a negatively charged S1 site and the combination of Ser441, Gly462 and Gly472 form a deep hydrophobic pocket to accommodate hydrophobic amino acids of a peptide substrate. The characteristic oxyanion hole of chymotrypsin family serine protease enzymes is formed by the backbone of Gly439 and Ser441. The polypeptide binding site is formed by residues 460–462 (Ser460, Trp461 and Gly462), which would be expected to form an antiparallel β-sheet with the backbone of the P1-P3 residues of a peptide substrate (Figure 7, Table S3 and Figure S9 in Supplementary Materials).

Having established the binding site and amino acids within the S1 pocket, docking was performed with ligands to generate TMPS2-ligand complexes.

### 2.4. Refinement of TMPS2 Homology Model after MDS and Docking Studies

The complexes were prepared by docking a database of ligands in the TMPS2-5CE1 homology model using MOE [44]. Using site finder, the active site chosen contained Ser441 and the majority of the S1 pocket amino acids: Glu389, Tyr416, Asp435, Ser436, Cys437, Gln438, Ser441, Thr459, Ser460, Trp461, Gly462, Ser463, Gly464, Cys465, Ala466, Arg470, Pro471, Gly472 and Val473.

**Figure 8:**
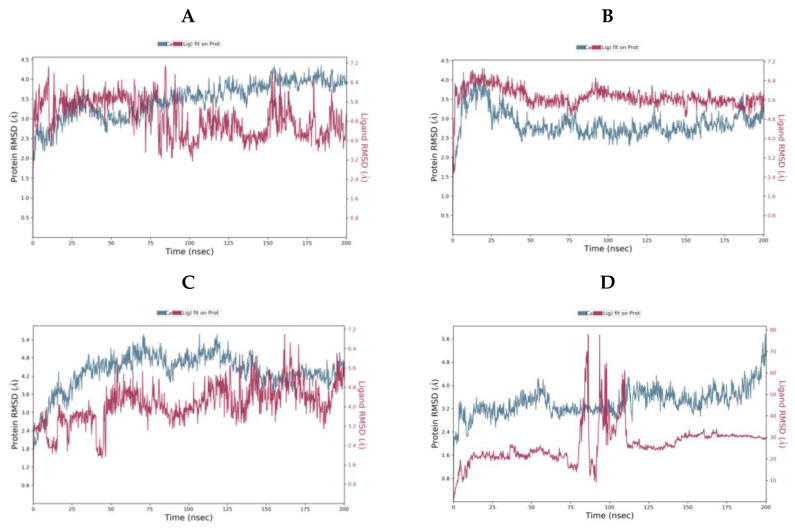
Protein-Ligand RMSD over the 200 ns MD simulation for (**A**) camostat (**B**) nafamostat (**C**) gabexate (**D**) sivelestat.

The ligand-protein complexes were subjected to 200 ns molecular dynamics (MD) simulations using the Desmond programme of Maestro (Figure 8).

For camostat, nafamostat and gabexate, the protein equilibrates. However, for sivelestat, the RMSD is still increasing indicating the protein is undergoing a significant conformational change at the end of the simulation. Likewise, the ligand RMSD for sivelestat is significantly larger than the RMSD of the protein indicating that the ligand has diffused away from the initial binding site. The most stable system is the nafamostat-TMPS2 complex, which equilibrates rapidly, while there are more fluctuations with camostat and gabexate (Table 4).

**Figure 9:**
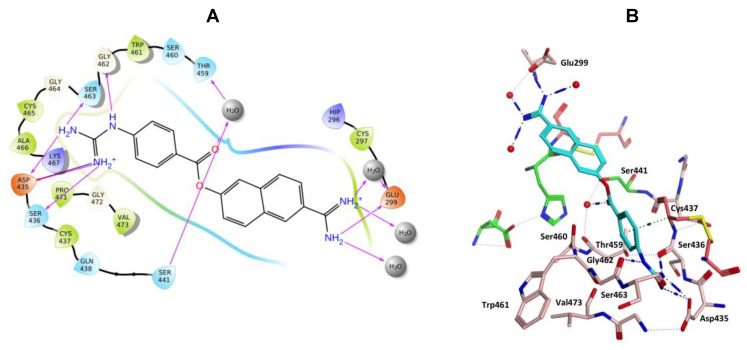
(**A**) 2D Ligand interactions and (**B**) 3D visualization of nafamostat (cyan).

Nafamostat was optimal with respect to binding, with the phenyl guanidine group situated within the S1 pocket through a salt bridge with Asp435 and H-bonding interactions with Asp435, Ser436, Ser463 and Gly462 (Figure 9). The phenyl ring sits in the hydrophobic pocket composed of Thr459, Ser460, Trp461 and Val473. The carbonyl oxygen of the ester linkage forms a water mediated H-bonding interactions with Ser441 and the amidine group forms direct and water mediated H-bonding interactions with Glu299 (Figure 9). Visualization using MOE also identified an aryl-H binding interaction between the phenyl ring of nafamostat and the backbone of Cys437 (Figure 9B). As previously reported [45], the catalytic steps for the cleavage of peptide-like bonds involve an acyl-enzyme intermediate formation between the substrate and Ser441 followed by the hydrolysis of the acyl-enzyme intermediate, releasing the cleaved substrate and restoring the active form of the enzyme. Nafamostat with its guanidinobenzoate moiety can inhibit TMPS2 by mimicking the natural substrates, where the ester group, resembling a peptide bond, can react with the catalytic serine forming the acyl-enzyme intermediate while the guanidinobenzoyl group becomes linked to the catalytic serine Ser441, rendering nafamostat an effective chemical inhibitor, which meets our findings after MDS.

**Figure 10:**
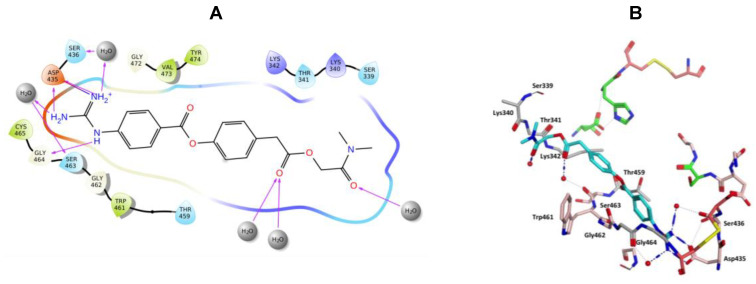
(**A**) 2D Ligand interactions and (**B**) 3D visualization of camostat (cyan).

**Figure 11:**
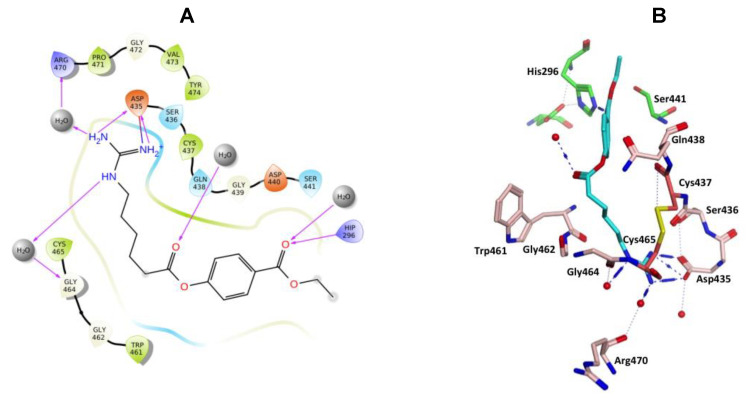
(**A**) 2D Ligand interactions and (**B**) 3D visualization of gabexate.

Camostat and gabexate were similar to nafamostat in the binding profiles of the guanidine group with the salt bridge formed with Asp435 and H-bonding interactions, direct or water mediated with Asp435, Ser436, Ser463 and Gly464 for camostat (Figure 10), and with Asp435, Gly464 and Arg470 for gabexate (Figure 11). Camostat formed H-bonding interaction between the ester and amide carbonyl oxygen atoms on the opposite side with water solvation molecules, while gabexate formed H-bonding interaction between the two ester carbonyl oxygens and water of solvation and the terminal ester also formed a H-bonding interaction with His296 (Figure 10 and Figure 11).

**Figure 12:**
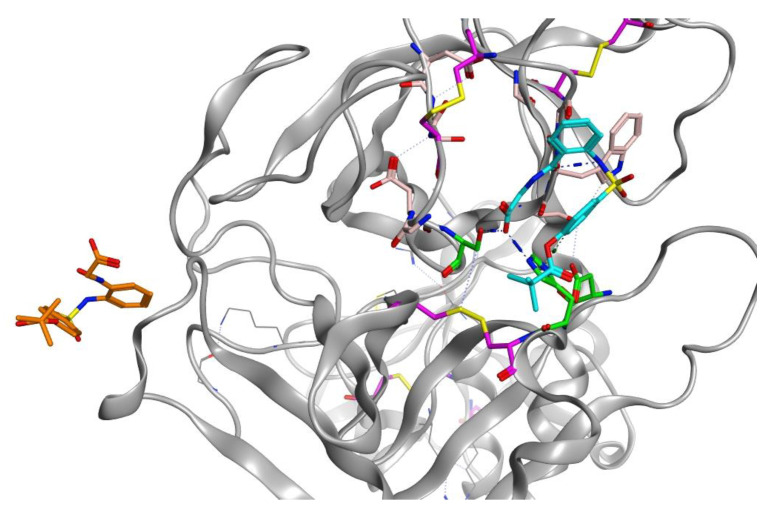
3D visualization of sivelestat showing the original position (cyan) in the S1 binding pocket and after 200 ns MD simulation (orange).

Sivelestat did not bind in the S1 binding site and, as indicated from the protein-ligand RMSD (Figure 8D), had moved away from the original site. Figure 12 shows the original position of sivelestat (cyan) compared with the final position (orange) after 200 ns MD simulation.

For the ligand-TMPS2 complexes of camostat, nafamostate and gabexate complexes, the mean ΔG (bind) was calculated [46] over two time frames, from 100–200 ns and the final 10 ns (190–200 ns) of the MD simulation (Table 5). The ΔG values indicate positioning within the TMPS2 protein was optimal for nafamostat, with ΔG values of −60.99 ± 4.27 and −61.47 ± 4.44 kcal/mol, respectively over the two timeframes. Thus, nafamostat would appear to have better binding affinity, which supports the observations from protein-ligand RMSD (Figure 8B) and interaction figures (Figure 9).

**Figure 13:**
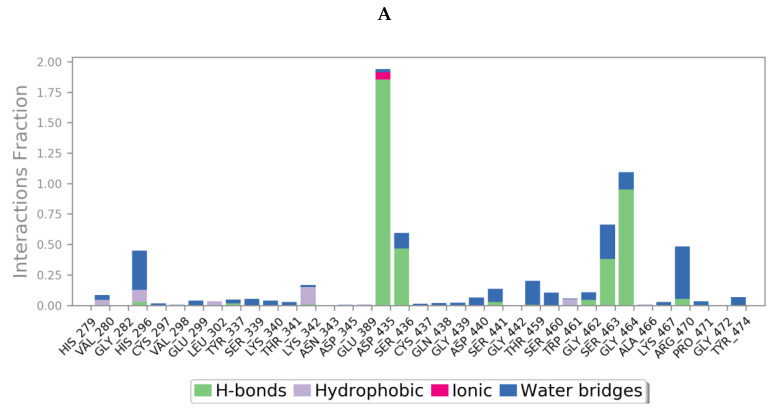

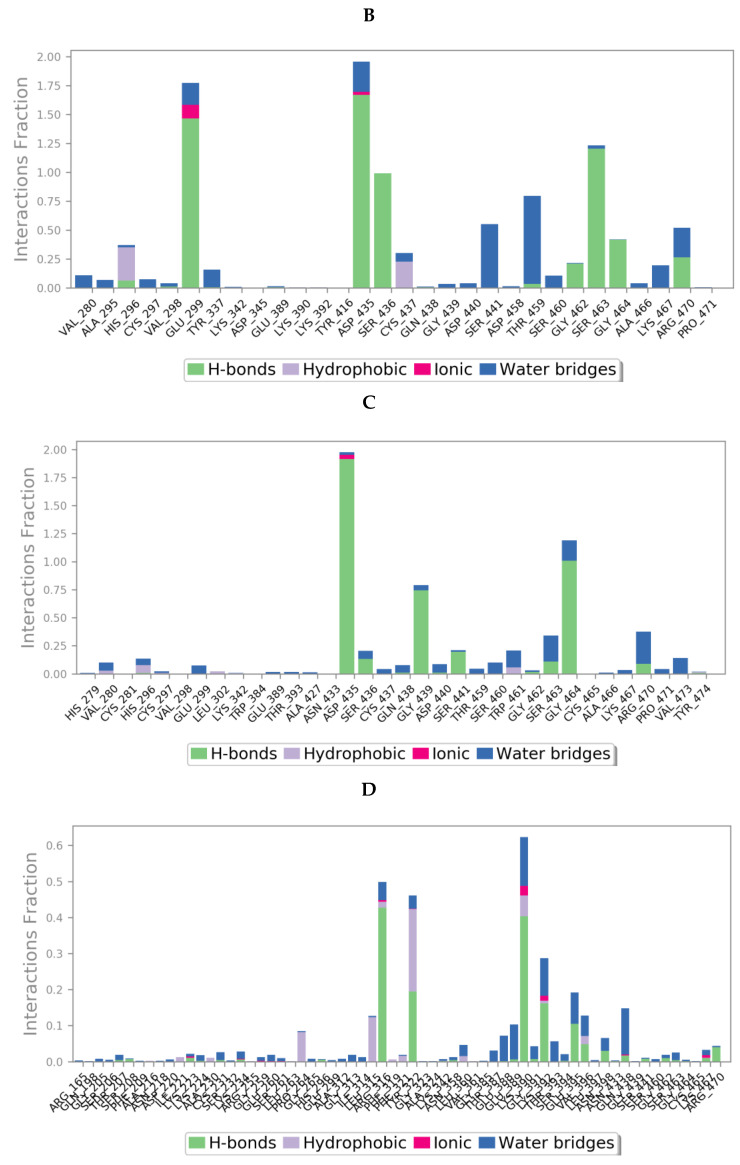
A schematic of detailed ligand atom interactions of (**A**) camostat, (**B**) nafamostat, (**C**) gabexate and (**D**) sivelestat with the protein residues of TMPS2 protein. Interactions that occur more than 30.0% of the simulation time in the selected trajectory (0 through 200 ns) are shown. [Hydrophobic (purple), water bridges (blue), H-bonds (green), ionic (pink)].

Protein interactions with the ligand could be categorized by type and summarized, as shown in the plots below (Figure 13), where the protein-ligand interactions (or ‘contacts’) are categorized into four types: hydrogen bonds, hydrophobic, ionic and water bridges. This can also be visualised from the ligand-protein contacts 2D summary over the course of the 200 ns simulation (Figure S10 in Supplementary Materials).

Limitations of this study:

Although MD is a robust computational method for the study of protein-ligand complexes, the results described here are theoretical, therefore experimental enzyme inhibition (IC_50_) assays, with the appropriate replicas, against TMPS2 are required to validate the theoretical findings.

## 3. Materials and Methods 

### 3.1. Transmembrane Protease Serine 2 (TMPS2_HUMAN) Molecular Modelling

The homologous sequences for building the 3D structure were searched using NCBI-BlastP (basic local alignment search tool). For alignments, BLOSUM62 substitution matrix was used as a comparison matrix. Clustal Omega from the European Bioinformatics Institute (EBI) was used to determine the conserved areas between the selected proteins and target protein TMPS2. SWISS-MODEL server accessible via ExPASy web server was used in comparative modelling of TMPS2. The constructed models were then evaluated.

### 3.2. Refinement and Evaluation of the Model

The Ramachandran plot was provided by the MolProbity server at Duke Biochemistry, US [37]. The PDB files of the homology models were uploaded. H-atoms were added, and the gaps were filled in the protein backbone with JiffiLoop (beta-test). The results were displayed and subdivided into regions of favoured, allowed, and disallowed [47]. The output data demonstrated a comparison between atom contacts, protein geometry, and peptide omegas in tables. A file of the best model in a PDB format was uploaded on ProSA (Protein Structure Analysis) [38] and the Z-score was calculated. Three parameters for each amino acid; secondary structure, degree of buried surface area, and fraction of side chain area that was covered by polar atoms were evaluated by Verify 3D service [39]. For each residue in the structure, these three parameters were evaluated, and a correlation was determined between the observed parameters and the ideal ones to which they were assigned.

### 3.3. Molecular Dynamic Simulation (MDS) and Docking Studies

Molecular docking and molecular dynamics simulations were performed as previously described [48]. Briefly docking studies using the TMPS2 homology model to generate PDB files of the TMPS2-ligand complexes were performed using MOE [44] until an RMSD gradient of 0.01 Kcal/mol/Å with the MMFF94 forcefield (ligands) and partial charges were automatically calculated. Docking was performed using the Alpha Triangle placement to determine the poses, refinement of the results was done using the MMFF94 forcefield, and rescoring of the refined results using the London ΔG scoring function was applied. The output database dock file was created with different poses for each ligand and arranged according to the final score function (S), which is the score of the last stage that was not set to zero.

Molecular dynamics simulations were run on the TMPS2-ligand complexes with the PDB files first optimised with protein preparation wizard in Maestro by assigning bond orders, adding hydrogen, and correcting incorrect bond types. A default quick relaxation protocol was used to minimise the MD systems with the Desmond programme. The orthorhombic water box allowed for a 10 Å buffer region between protein atoms and box sides. Overlapping water molecules were deleted, and the systems were neutralised with Na^+^ ions and salt concentration 0.15 M. Force-field parameters for the complexes were assigned using the OPLS_ 2005 forcefield, that is, a 200 ns molecular dynamic run in the NPT ensemble (T = 300 K) at a constant pressure of 1 bar. Energy and trajectory atomic coordinate data were recorded at each 1.2 ns. Prime/MMGBAS, available in Schrödinger Prime suite, was used to calculate the binding free energy of the ligands with TMPS2.

ΔG (bind) = E_complex (minimised) − (E_ligand (minimised) + E_receptor (minimised))

Two mean ΔG (bind) values were calculated from (1) each 5 frames of the final 100 ns, and (2) each frame of the final 10 ns of the MD simulation. The average generated ΔG was from each energy minimised frame using the equation shown above.

## 4. Conclusions

In this study, a high-quality homology model of the 3D human TMPS2 was constructed to enable investigation of TMPS2 as a promising drug target for the development of therapeutics in the treatment of SARS-CoV2. Molecular dynamics simulations were performed, and the active site conserved amino acids were selected as a docking site for camostat, nafamostat, gabexate, and sivelestat. Quite clearly nafamostat has optimal binding owing to its ability to anchor both ends of its structure through a salt bridge with Asp345 and the guanidine group, and H-bonding with Glu299 and the amidine group. Camostat and gabexate are able to anchor the guanidine group effectively via salt bridge formation with Asp345, however the dimethylamide of camostat is not able to form an anchor with the protein, while gabexate, which is a more flexible molecule, was able to form a H-bonding interaction with His296. Computational binding affinity (ΔG) studies support the findings of the MD studies and the requirement of the basic guanidine moiety, or equivalent functional group, in the salt bridge formation with Asp345. Sivelestat, which lacks a basic functional group, is unable to form a tether with Asp435 and rather diffuses from the S1 site to a position where the acidic carboxylate group of sivelestat can form a salt bridge with a basic amino acid, Arg316 (Figure S11 in Supplementary Materials).

The studies also indicate the preference for a basic moiety at the other end of the inhibitor to form an ionic bond with an acidic amino acid and secure the inhibitor within the S1 binding pocket as observed with nafamostat with an amidine group. The ability of gabexate to tether the other end of its structure through a H-bonding interaction provides some scope to investigate additional H-bond forming functional groups. In addition, the optimal length and flexibility of a TMPS2 inhibitor ligand to fit and bind within the S1 pocket would warrant further investigation through inhibitor design.

## Figures and Tables

**Table 1:** Initial screening for possible templates using BLAST and SWISS-MODEL analyses against the PDB resolved structures.

PDB Code	Protein	Query Cover	E-Value	Percent Identity
5CE1_A	TMPS1	94%	7e–67	33.61
1Z8G_A	TMPS1	69%	4e–62	33.52
6KD5_B	TMPS13	47%	3e–64	45.19
6O1G_A	Plasma kallikrein	51%	2e–61	42.21

**Table 2:** Validation data for the four TMPS2 homology models.

Model	Ramachandran Outliers	ProSA Z-Score	Verify3D
Model 1 (5CE1_A template)	3 (Ser208, Ala216, Arg255)	−8.67	95.38%
Model 2 (1Z8G_A template)	3 (Ser215, Leu248, Val415)	−8.76	91.59%
Model 3 (6KD5_B template)	2 (Pro301, Asn433)	−6.80	97.02%
Model 4 (6O1G_A template)	5 (Asn303, Asn304, Phe321, Pro305, Pro369)	−6.57	91.56%

**Table 3:** Active site and domains of the most homologous TMPS proteins.

				Domains			
Protein	Uniprot ID	Active Site His/Asp/Ser	Cleavage Site	LDL-Receptor Class A	SRCR	Serine Peptidase	X-ray Structure
TMPS1	P05981	203/257/353	162–163	-	54–151	163–405	5CE1, 1Z8G, 1P57
TMPS2	O15393	296/345/441	255–256	112–149	150–242	256–489	-
TMPS3	P57727	257/304/401	216–217	72–108	109–205	217–449	-
TMPS4	Q9NRS4	245/290/387	204–205	61–93	94–204	205–434	-
TMPS5	Q9H353	258/308/405	217–218	-	112–207	218–453	-
TMPS13	Q9BYE2	366/414/511	325–326	204–226	195–325	326–559	6KD5

SRCR = scavenger receptor cysteine rich; LDL = low-density lipoprotein.

**Table 4:** Protein-ligand RMSD (Å) at 0 and 200 ns.

	RMSD (Å) 0 ns		RMSD (Å) 200 ns	
Ligand-Complex	Protein	Ligand	Protein	Ligand
Camostat	2.18	5.32	3.90	5.05
Nafamostat	1.83	4.85	3.21	5.83
Gabexate	2.20	2.98	4.35	5.87
Sivelestat	2.25	2.21	5.07	29.42

**Table 5:** Computational binding affinity (ΔG) of the ligand-protein complexes.

Complex	ΔG (kcal/mol) 100–200 ns	ΔG (kcal/mol) 190–200 ns
Camostat-TMPS2	−48.87 ± 5.07	−46.24 ± 4.42
Nafamostat-TMPS2	−60.99 ± 4.27	−61.47 ± 4.44
Gabexate-TMPS2	−54.37 ± 4.86	−51.91 ± 4.86

## Supplementary Materials

Table S1: Stereochemical quality assessment of TMPS2 model 1 (5CE1_A template), Table S2: Stereochemical quality assessment of TMPS2 model 2 (1Z8G_A template), Table S3: Comparison between TMPS2 and the template 5CE1_A, Figure S1: Ramachandran plot for TMPS2 model 1 (5CE1_A template); 92.2% (317/344) of all residues were in favoured (98%) regions, 99.1% (341/344) of all residues were in allowed (>99.8%) regions. There were 3 outliers (phi, psi): 208 Ser (−58.3, −174.6), 216 Ala (−25.8, −45.7) and 255 Arg (88.3, 25.7), Figure S2: Ramachandran plot for TMPS2 model 2 (1Z8G_A template); 94.2% (323/343) of all residues were in favoured (98%) regions, 99.1% (340/343) of all residues were in allowed (>99.8%) regions. There were 3 outliers (phi, psi): 215 Ser (−56.0, 2.4), 248 Leu (−50.9, −74.7) and 415 Val (−104.8, −94.8), Figure S3: Z-scores of TMPS2 model 1 (5CE1_A template) determined by X-ray crystallography (light blue) or NMR spectroscopy (dark blue), and local model quality by plotting energies as a function of amino acid sequence position, a positive value indicates erroneous parts; most of the amino acids gave negative values, Figure S4: Z-scores of TMPS2 model 2 (1Z8G_A template) determined by X-ray crystallography (light blue) or NMR spectroscopy (dark blue), and local model quality by plotting energies as a function of amino acid sequence position, a positive value indicates erroneous parts; most of the amino acids gave negative values, Figure S5: Verify 3D results for model 1 (5CE1_A template) showing that the model passes in the 3D/1D profile, Figure S6: Verify 3D results for model 2 (1Z8G_A template) showing that the model passes in the 3D/1D profile, Figure S7: Overlap of 5CE1_A (blue) and 1Z8G_A (yellow) after MD simulation, Figure S8: Sequence alignment of TMPS enzymes using Clustal O in which "∗" means that the residues are identical, ":" means that conserved substitutions have been observed, "." means that semi-conserved substitutions are observed. The SRCR domain is indicated in blue lettering, cysteine residues involved in disulphide bond formation are highlighted in yellow, the catalytic triad residues are highlighted in cyan and the cleavage site is highlighted in pink, Figure S9: Comparison between the secondary structure of TMPS2 and the template 5CE1_A after alignment and superimposition using MOE software, showing α-helices as red lines, β-sheets as yellow arrows and loops as blue lines, Figure S10: A schematic of detailed ligand atom interactions of (A) camostat, (B) nafamostat, (c) gabexate and (D) sivelestat with the protein residues of TMPS2 protein. Interactions that occur more than 30.0% of the simulation time in the selected trajectory (0 through 200 ns) are shown, Figure S11: 2D Ligand interactions of sivelestat in TMPS2 protein.
