# Supplementary material for: Molecular Insights into Human Transmembrane Protease Serine-2 (TMPS2) Inhibitors against SARS-CoV2: Homology Modelling, Molecular Dynamics, and Docking Studies
Source: Molecules. 2020 Oct 29;25(21):5007. doi: 10.3390/molecules25215007 (PMC7663346; doi:10.3390/molecules25215007)
Supplement: Supplementary file 1 [file molecules-25-05007-s001.pdf]

Supplementary materials

Table S1. Stereochemical quality assessment of TMPS2 model 1 (5CE1_A template).

Protein Geometry	Poor rotamers	3	1.02%	Goal: <0.3%
	Favoured rotamers	278	94.24%	Goal: >98%
	Ramachandran outliers	3	0.87%	Goal: <0.05%
	Ramachandran favoured	317	92.15%	Goal: >98%
	Rama distribution Z-score	-1.49 ± 0.44		Goal: abs(Z score) < 2
	Cβ deviations >0.25Å	6	1.89%	Goal: 0
	Bad bonds:	0 / 2779	0.00%	Goal: 0%
	Bad angles:	31 / 3779	0.82%	Goal: <0.1%
Peptide Omegas	Cis Prolines:	0 / 16	0.00%	Expected: ≤1 per chain, or ≤5%
Low-resolution Criteria	CaBLAM outliers	7	2.0%	Goal: <1.0%
	CA Geometry outliers	4	1.17%	Goal: <0.5%

Table S2. Stereochemical quality assessment of TMPS2 model 2 (1Z8G_A template).

Protein Geometry	Poor rotamers	4	1.36%	Goal: <0.3%
	Favoured rotamers	283	96.26%	Goal: >98%
	Ramachandran outliers	3	0.87%	Goal: <0.05%
	Ramachandran favoured	323	94.17%	Goal: >98%
	Rama distribution Z-score	-1.31 ± 0.44		Goal: abs(Z score) < 2
	Cβ deviations >0.25Å	2	0.63%	Goal: 0
	Bad bonds:	2 / 2771	0.07%	Goal: 0%
	Bad angles:	22 / 3768	0.58%	Goal: <0.1%
Peptide Omegas	Cis Prolines:	0 / 16	0.00%	Expected: ≤1 per chain, or ≤5%
Low-resolution Criteria	CaBLAM outliers	8	2.3%	Goal: <1.0%
	CA Geometry outliers	5	1.47%	Goal: <0.5%

Table S3. Comparison between TMPS2 and the template 5CE1_A

Secondary structures	Template 5CE1_A		TMPS2 model	
	Residues	Total	Residues	Total
**Helices**	Ala38-Met48<br>Ala157-Cys159<br>Leu167-Ser168<br>Gln274-Cys277<br>Val348-Thr361	34 amino acids (9.14%)	Lys83-Met109<br>Glu178-Tyr103<br>Arg182-Asp187<br>Asp220- Lys224<br>Ala295-Cys297<br>Thr407-Cys410<br>Val477-Ala490	62 amino acids (12.60%)
**Sheets**	Val9-Ser12<br>Arg17-Asp22<br>Trp28-Cys32<br>Leu54-Val61<br>Ser70-Val75<br>Gln84-Asp94<br>Phe100-Cys105<br>Arg122-Asp123<br>Gln132-Tyr137<br>Ala140-Ser149<br>Trp152-Thr155<br>Arg169-Ala178<br>Leu185-Tyr194<br>Ala214-Leu218<br>Ile245-Gly250<br>Gln265-Val269<br>Ile271-Ile272<br>Met291-Ala294<br>Pro311-Glu315<br>Trp324-Trp332<br>Gly343-Lys347	129 amino acids (34.68%)	Val149-Tyr152<br>Ile157-Ser162<br>Trp168-His169<br>Lys195-Val201<br>Phe209-Asn213<br>Leu225-Tyr226<br>Val236-Cys241<br>Ile256-Val257<br>Glu260-Ser261<br>Gln270-Val275<br>Val278-Ile286<br>Trp290-Thr293<br>Trp308-Arg316<br>Gly325-Ser333<br>Ala347-Leu351<br>Leu378-Gly383<br>Asn398-Ile405<br>Met424-Ala427<br>Pro444-Lys449<br>Ile452-Ser460<br>Gly472-Asn476	116 amino acids (23.58%)
**Loops**	Ser1-Pro8<br>Ser13-Ala16<br>Lys23-Thr27<br>Ser33-Gln37<br>Gly45-Ala53<br>Arg62-Thr69<br>Asp76-Thr83<br>Cys95-Arg99<br>Gln106-Gly121<br>Thr124-Trp131<br>Asp138-Gly139<br>Gly150-Asp151<br>Ala156<br>Phe160-Val166<br>Gln179-Gly184<br>His195-Ile213<br>Ser219-Lys244<br>Trp251-Leu264<br>Pro270<br>Ser273<br>Asn278-Lys290<br>Gly295-Gly300<br>Ile301-Gly310<br>Asp316-Arg323<br>Gly333-Pro342<br>His362-Leu372	209 amino acids (56.18%)	Met1-Lys82<br>Gly110-Cys148<br>Gly153-Phe156<br>Ser163-Ser167<br>Pro170-Asn177<br>Gly181<br>Met188-Phe194<br>Asp202-Ser208<br>Thr214-Val219<br>His227-Ala235<br>Ile242-Arg255<br>Gly258-Gly259<br>Ala262-Trp269<br>Gln276-Asn277<br>Thr287-Glu289<br>Ala294<br>Val298-His307<br>Gln317-Ala324<br>His334-Ile346<br>Gln352-Gln377<br>Trp385-Leu397<br>Glu406<br>Asn411-Ala423<br>Gly428-Gly443<br>Asn450-Asn451<br>Trp461-Pro471<br>Asp491-Gly492	314 amino acids (63.82%)

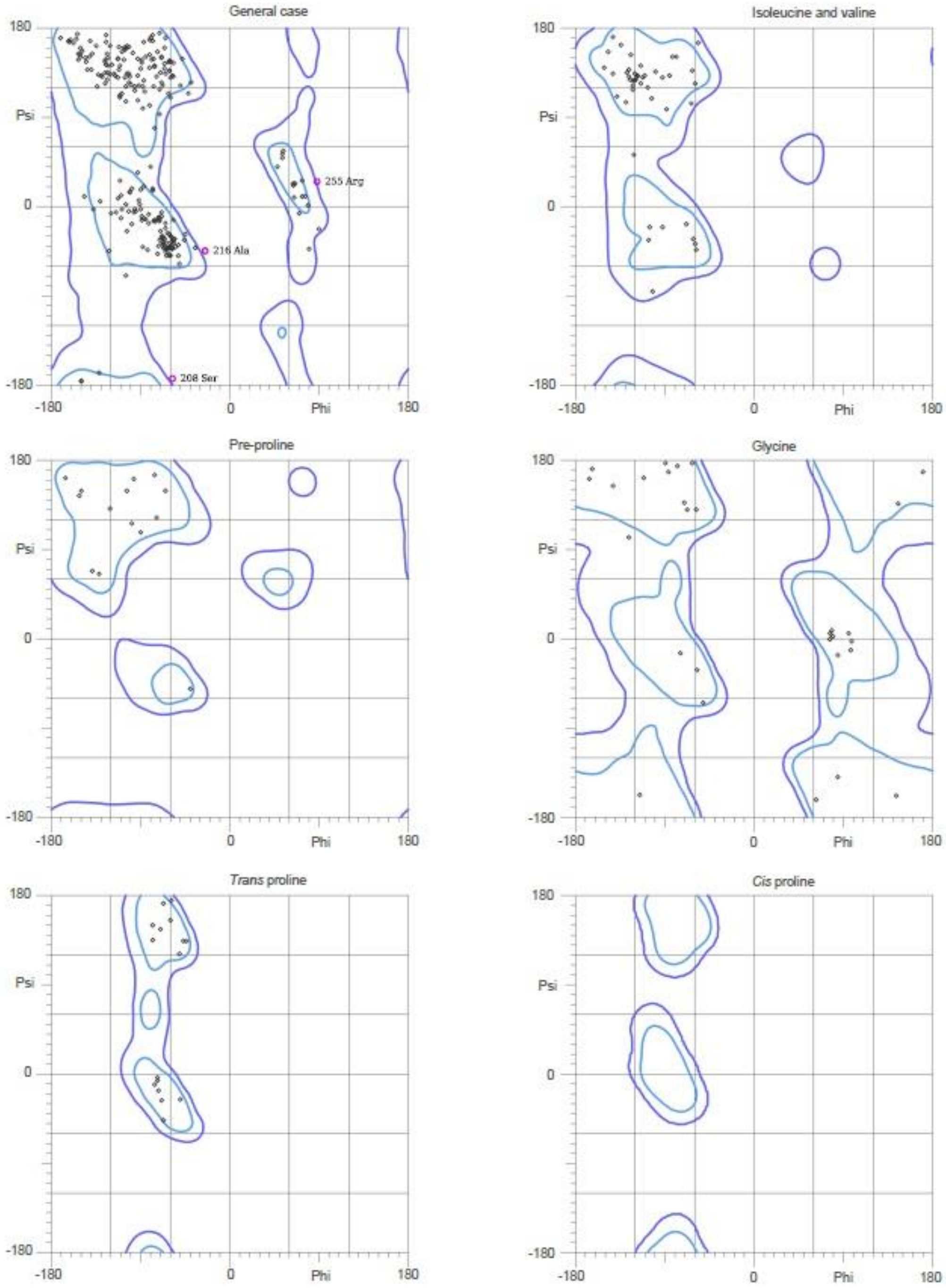

Figure S1. Ramachandran plot for TMPS2 model 1 (5CE1_A template); 92.2% (317/344) of all residues were in favoured (98%) regions, 99.1% (341/344) of all residues were in allowed (>99.8%) regions. There were 3 outliers (phi, psi): 208 Ser (-58.3, -174.6), 216 Ala (-25.8, -45.7) and 255 Arg (88.3, 25.7).

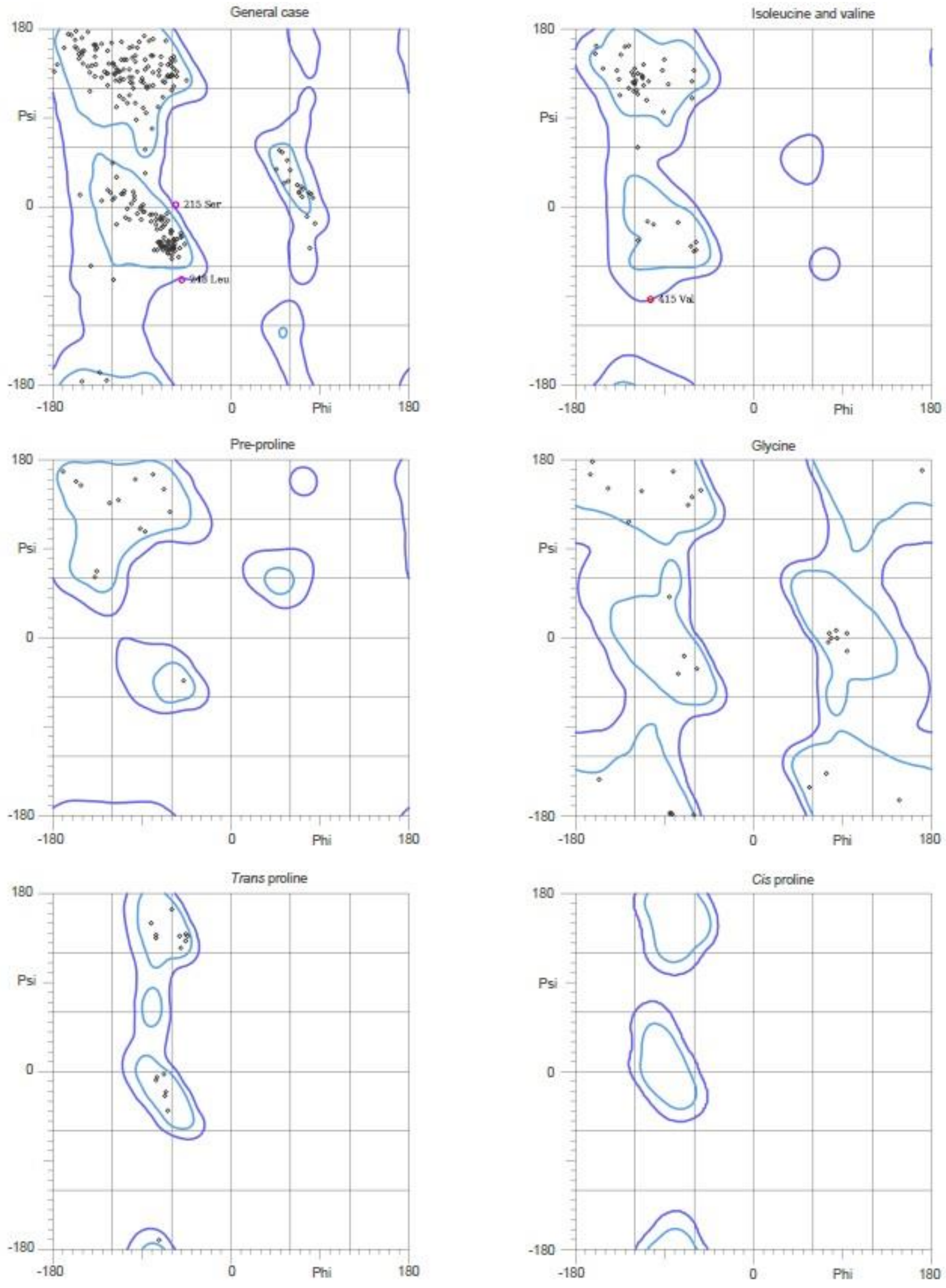

Figure S2. Ramachandran plot for TMPS2 model 2 (1Z8G_A template); 94.2% (323/343) of all residues were in favoured (98%) regions, 99.1% (340/343) of all residues were in allowed (>99.8%) regions. There were 3 outliers (phi, psi): 215 Ser (-56.0, 2.4), 248 Leu (-50.9, -74.7) and 415 Val (-104.8, -94.8).

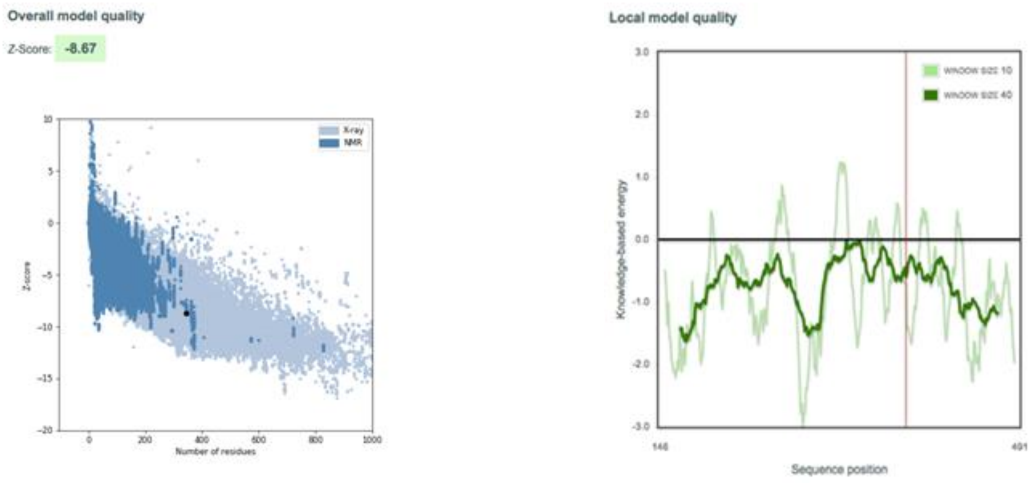

Figure S3. Z-scores of TMPS2 model 1 (5CE1_A template) determined by X-ray crystallography (light blue) or NMR spectroscopy (dark blue), and local model quality by plotting energies as a function of amino acid sequence position, a positive value indicates erroneous parts; most of the amino acids gave negative values.

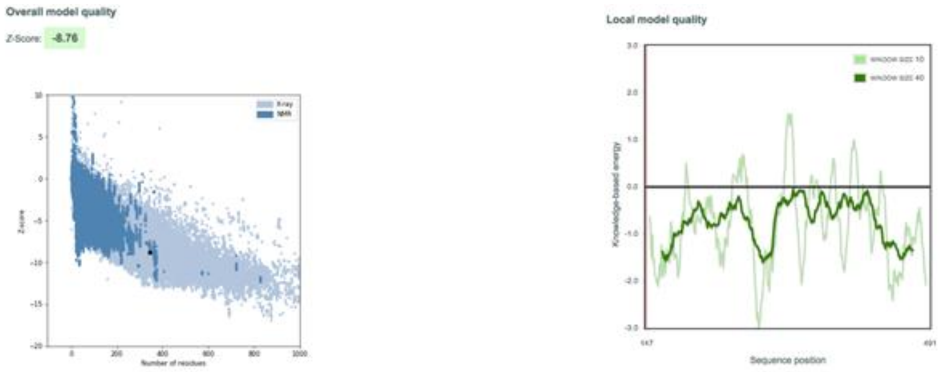

Figure S4. Z-scores of TMPS2 model 2 (1Z8G_A template) determined by X-ray crystallography (light blue) or NMR spectroscopy (dark blue), and local model quality by plotting energies as a function of amino acid sequence position, a positive value indicates erroneous parts; most of the amino acids gave negative values.

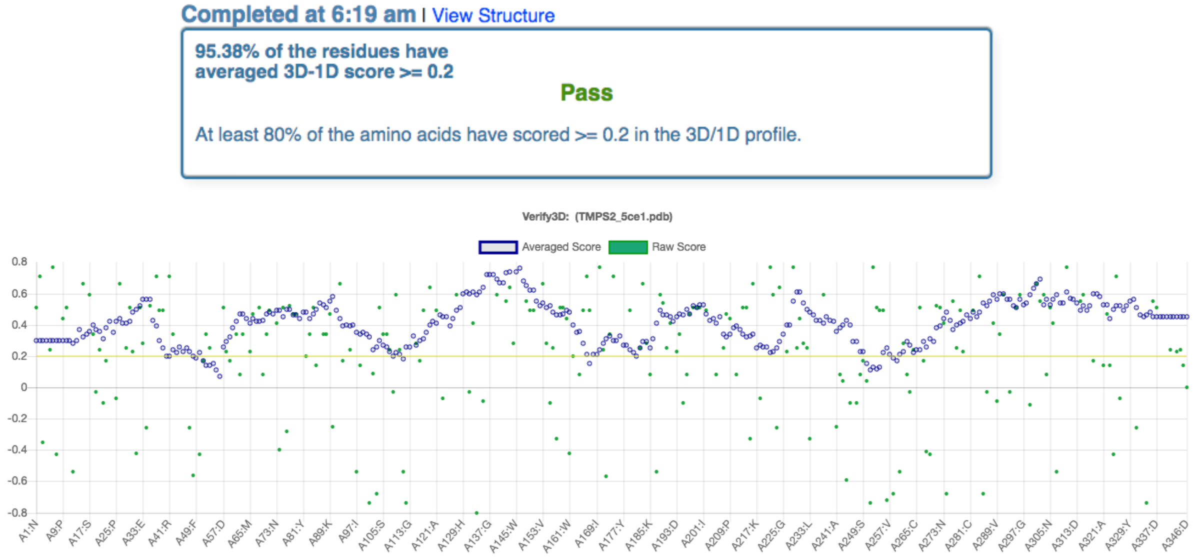

Figure S5. Verify 3D results for model 1 (5CE1_A template) showing that the model passes in the 3D/1D profile.

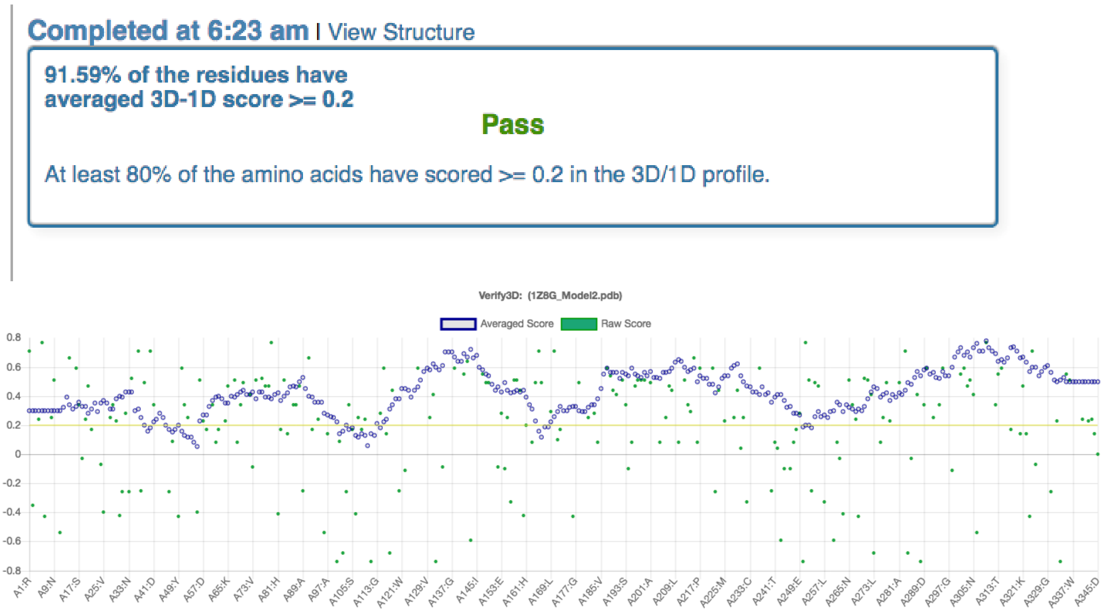

Figure S6. Verify 3D results for model 2 (1Z8G_A template) showing that the model passes in the 3D/1D profile.

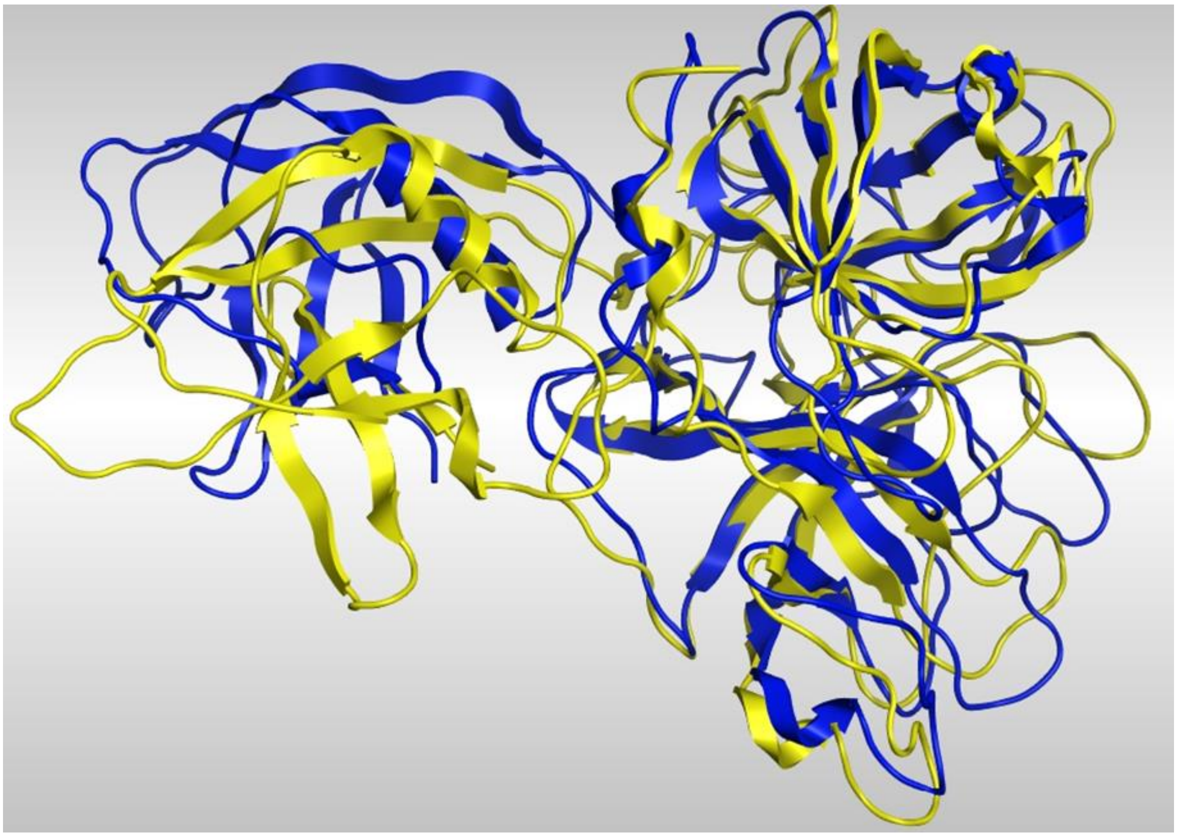

Figure S7. Overlap of 5CE1_A (blue) and 1Z8G_A (yellow) after MD simulation.

```
CLUSTAL O(1.2.4) multiple sequence alignment
sp|P05981|TMPS1_HUMAN     ------------------------------------------------------------0
sp|Q9NRS4|TMPS4_HUMAN     ------------------------------------------------------------0
sp|Q9H3S3|TMPS5_HUMAN     -----------------------------------------MSLMLDDQPPMEAQ-----14
sp|Q9BYE2|TMPS13_HUMAN    MERDSHGNASPARTPSAGASPAQASPAGTPPGRASPAQASPAQASPAGTPPGRASPAQAS60
sp|O15393|TMPS2_HUMAN     ------------------------------------------MALNSGSPPAIGPYYE--16
sp|P57727|TMPS3_HUMAN     --------------------------------------------MGENDPPAVEAPFS--14


sp|P05981|TMPS1_HUMAN     ------------------------------------------------------------0
sp|Q9NRS4|TMPS4_HUMAN     ------------------------------------------------------------0
sp|Q9H3S3|TMPS5_HUMAN     ------------------------------------------------------------14
sp|Q9BYE2|TMPS13_HUMAN    PAGTPPGRASPGRASPAQASPAQASPARASPALASLSRSSSGRSSSARSASVTTSPTRVY120
sp|O15393|TMPS2_HUMAN     NHGYQP----------ENPYPAQP----------TVVPTVYE----VHPAQYYPSPVPQY52
sp|P57727|TMPS3_HUMAN     ------------------------------------FRSLFG----LDDLKI--------26


sp|P05981|TMPS1_HUMAN     -------------------------MAQKEGG---RT--VPCCSRPKVAALTAGTLLLLT30
sp|Q9NRS4|TMPS4_HUMAN     -----------MLQDPDSDQPL-NSLDVKPL--RKPRIPMETFRKVG--IPIIIALLSLA44
sp|Q9H3S3|TMPS5_HUMAN     ---------------YAEEGPGPGIFRAEPGDQQHPISQAVCWRSMRRGCAVLGALGLLA59
sp|Q9BYE2|TMPS13_HUMAN    LVRATPVGAVPIRSSPARSAPATRATRESPG----TSLPKFTWREGQKQLPLIGCVLLLI176
sp|O15393|TMPS2_HUMAN     APRVLTQASNPVVCTQPK-SPSGTVCTSKTK--------K-----ALCITLTLGTFLVGA98
sp|P57727|TMPS3_HUMAN     ---------------SPV-APDADAVAAQIL----SLLPL-----KFFPIIVIGIIALIL61
                                                      .                          .

sp|P05981|TMPS1_HUMAN     AIGAAS-WAIVAVLLRS-------------------------------------------46
sp|Q9NRS4|TMPS4_HUMAN     SI----IIVVVLIKVILDKYY-----FLCGQPLHFIPRKQLCDGELDCPLGEDEEHCVKS95
sp|Q9H3S3|TMPS5_HUMAN     GAGVGS-WLLVLYLCPAASQPISGTLQDEEITLSCSEASAEEAL----LPALPKTVSFR-113
sp|Q9BYE2|TMPS13_HUMAN    ALVVSLIILFQFWQGHTGIRY-------KEQRESCPKHAVRCDGVVDCKLKSDELGCVR-228
sp|O15393|TMPS2_HUMAN     ALAAGLLWKFMGSKCSNSGIE-------CDSSGTCINPSNWCDGVSHCPGGEDENRCVR-150
sp|P57727|TMPS3_HUMAN     ALAIGLGIHF---DC-SGKYR-------CRSSFKCIELIARCDGVSDCKDGEDEYRCVR-109
                          .        .

sp|P05981|TMPS1_HUMAN     -DQEPLYPVQVSSADARLMVFDKTEGTWRLLCSSRSNARVAGLSCEEMGFLRALTHSELD105
sp|Q9NRS4|TMPS4_HUMAN     FPEGPAVAVRLSKDRSTLQVLDSATGNWFSACFDNFTEALAETACRQMGYSSKPTFRAVE155
sp|Q9H3S3|TMPS5_HUMAN     ----------INSEDFLLEAQVRDQPRWLLVCHEGWSPALGLQICWSLGHLRLTHHKGVN163
sp|Q9BYE2|TMPS13_HUMAN    ----------FDWDKSLLKIYSGSSHQWLPICSSNWNDSYSEKTCQQLGFESAHRTTEVA278
sp|O15393|TMPS2_HUMAN     ----------LYGPNFILQVYSSQRKSWHPVCQDDWNENYGRAACRDMGYKNNFYSSQGI200
sp|P57727|TMPS3_HUMAN     ----------VGGQNAVLQVFTA--ASWKTMCSDDWKGHYANVACAQLGFPSYVSSDNLR157
                                    .      *         *   * .  .   .   * .:*.

sp|P05981|TMPS1_HUMAN     VRTAGANGTSGFFCVDEGRL---PHTQRLLEVISVCDCPRGRFLAAICQDCGRRK--LPV160
sp|Q9NRS4|TMPS4_HUMAN     IGPD-----QDLDVVEI-----TENSQELRMRNSSGPCLSGSLVSLHCLACGKSL---KT202
sp|Q9H3S3|TMPS5_HUMAN     LTDIKLNSSQEFAQLSP-----RLGGFLEEAWQPRNNCTSGQVVSLRCSECGARP---LA215
sp|Q9BYE2|TMPS13_HUMAN    HRDF----ANSFSIL-------RYNS-TIQESLHRSECPSQRYISLQCSHCGLRA---MT323
sp|O15393|TMPS2_HUMAN     VDDS---GSTSFMK---LNTSAGNVD-IYKKLYHSDACSSKAVVSLRCIACGVNLNSSRQ253
sp|P57727|TMPS3_HUMAN     VSSLEGQFREEFVSIDHLLPDDKVTA-LHHSVYVREGCASGHVVTLQCTACGHRR--GYS214
                                     :                         *     ::  *  **

sp|P05981|TMPS1_HUMAN     DRIVGGRDTSLGRWPWQVSLRYDGAHLCGGSLLSGDWVLTAAHCFPER-NRVLSRWRVFA219
sp|Q9NRS4|TMPS4_HUMAN     PRVVGVEEASVDSWPWQVSIQYDKQHVCGGSILDPHWVLTAAHCFRKHT--DVFNWKVRA260
sp|Q9H3S3|TMPS5_HUMAN     SRIVGGQSVAPGRWPWQASVALGFRHTCGGSVLAPRWVVTAAHCMHSFRLARLSSWRVHA275
sp|Q9BYE2|TMPS13_HUMAN    GRIVGGALASDSKWPWQVSLHFGTTHICGGTLIDAQWVLTAAHCFFVTREKVLEGWKVYA383
sp|O15393|TMPS2_HUMAN     SRIVGGESALPGAWPWQVSLHVQNVHVCGGSIITPEWIVTAAHCVEKPLN-NPWHWTAFA312
sp|P57727|TMPS3_HUMAN     SRIVGGNMSLLSQWPWQASLQFQGYHLCGGSVITPLWIITAAHCVYDL-Y-LPKSWTIQV272
                           *:**      . ****.*:     * ***:::   *::*****.          *   .

sp|P05981|TMPS1_HUMAN     GAVAQASP-HGLQLGVQAVVYHGGYLPFRDPNSEENSNDIALVHLSSPLPLTEYIQPVCL278
sp|Q9NRS4|TMPS4_HUMAN     GSDKLGSF---PSLAVAKIIIIEFNP------MYPKDNDIALMKLQFPLTFSGTVRPICL311
sp|Q9H3S3|TMPS5_HUMAN     GLVSHSAVRPHQGALVERIIPHPLYS------AQNHDYDVALLRLQTALNFSDTVGAVCL329
sp|Q9BYE2|TMPS13_HUMAN    GTSNLHQ--LPEAASIAEIIINSNYT------DEEDDYDIALMRLSKPLTLSAHIHPACL435
sp|O15393|TMPS2_HUMAN     GILRQSFMFYGAGYQVEKVISHPNYD------SKTKNNDIALMKLQKPLTFNDLVKPVCL366
sp|P57727|TMPS3_HUMAN     GLVSL-LDNPAPSHLVEKIVYHSKYK------PKRLGNDIALMKLAGPLTFNEMIQPVCL325
                          *              :  ::                . *:**::*   * :.  :   **

sp|P05981|TMPS1_HUMAN     PAAGQALVDGKICTVTGWGNTQYY-GQQAGVLQEARVPIISNDVCNGADFYGNQIKPKMF337
sp|Q9NRS4|TMPS4_HUMAN     PFFDEELTPATPLWIIGWGFTKQNGGKMSDILLQASVQVIDSTRCNADDAYQGEVTEKMM371
sp|Q9H3S3|TMPS5_HUMAN     PAKEQHFPKGSRCWVSGWGHTHPSHTYSSDMLQDTVVPLFSTQLCNSSCVYSGALTPRML389
sp|Q9BYE2|TMPS13_HUMAN    PMHGQTFSLNETCWITGFGKTRETDDKTSPFLREVQVNLIDFKKCNDYLVYDSYLTPRMM495
sp|O15393|TMPS2_HUMAN     PNPGMMLQPEQLCWISGWGATEEK-GKTSEVLNAAKVLLIETQRCNSRYVYDNLITPAMI425
sp|P57727|TMPS3_HUMAN     PNSEENFPDGKVCWTSGWGATEDGAGDASPVLNHAAVPLISNKICNHRDVYGGIISPSML385
                          *     :         *:* *.      : .*  . * ::.   **    * . :.  *:

sp|P05981|TMPS1_HUMAN     CAGYPEGGIDACQGDSGGPFVCEDSISRTPRWRLCGIVSWGTGCALAQKPGVYTKVSDFR397
sp|Q9NRS4|TMPS4_HUMAN     CAGIPEGGVDTCQGDSGGPLMYQ-S----DQWHVVGIVSWGYGCGGPSTPGVYTKVSAYL426
sp|Q9H3S3|TMPS5_HUMAN     CAGYLDGRADACQGDSGGPLVCPDG----DTWRLVGVVSWGRGCAEPNHPGVYAKVAEFL445
sp|Q9BYE2|TMPS13_HUMAN    CAGDLRGGRDSCQGDSGGPLVCEQN----NRWYLAGVTSWGTGCGQRNKPGVYTKVTEVL551
sp|O15393|TMPS2_HUMAN     CAGFLQGNVDSCQGDSGGPLVTSKN----NIWWLIGDTSWGSGCAKAYRPGVYGNVMVFT481
sp|P57727|TMPS3_HUMAN     CAGYLTGGVDSCQGDSGGPLVCQER----RLWKLVGATSFGIGCAEVNKPGVYTRVTSFL441
                          ***   *  *:********::          * : * .*:* **.    **** .*

sp|P05981|TMPS1_HUMAN     EWIFQAIKTHSEASGMVTQL---------------   417
sp|Q9NRS4|TMPS4_HUMAN     NWIYNVWKAEL------------------------   437
sp|Q9H3S3|TMPS5_HUMAN     DWIHDTAQDSLL-----------------------   457
sp|Q9BYE2|TMPS13_HUMAN    PWIYSKMEVRSLQQDTAPSRLGTSSGGDPGGAPRL   586
sp|O15393|TMPS2_HUMAN     DWIYRQMRADG------------------------   492
sp|P57727|TMPS3_HUMAN     DWIHEQMERDLKT----------------------   454
```

Figure S8. Sequence alignment of TMPS enzymes using Clustal O in which "*" means that the residues are identical, ":" means that conserved substitutions have been observed, "." means that semi-conserved substitutions are observed. The SRCR domain is indicated in blue lettering, cysteine residues involved in disulphide bond formation are highlighted in yellow, the catalytic triad residues are highlighted in cyan and the cleavage site is highlighted in pink.

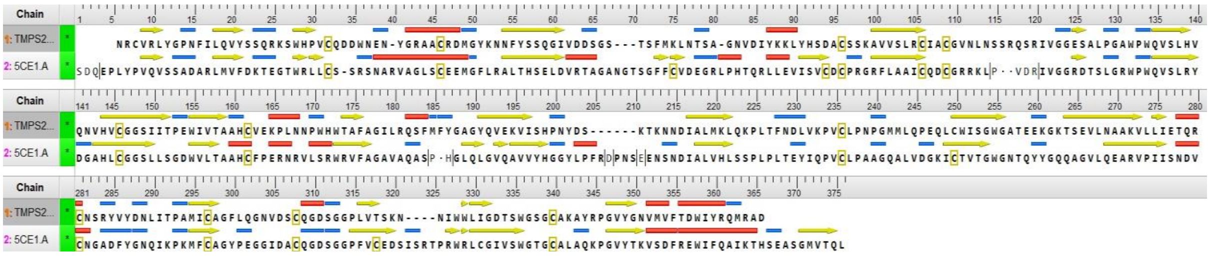

Figure S9. Comparison between the secondary structure of TMPS2 and the template 5CE1_A after alignment and superimposition using MOE software, showing α-helices as red lines, β-sheets as yellow arrows and loops as blue lines.

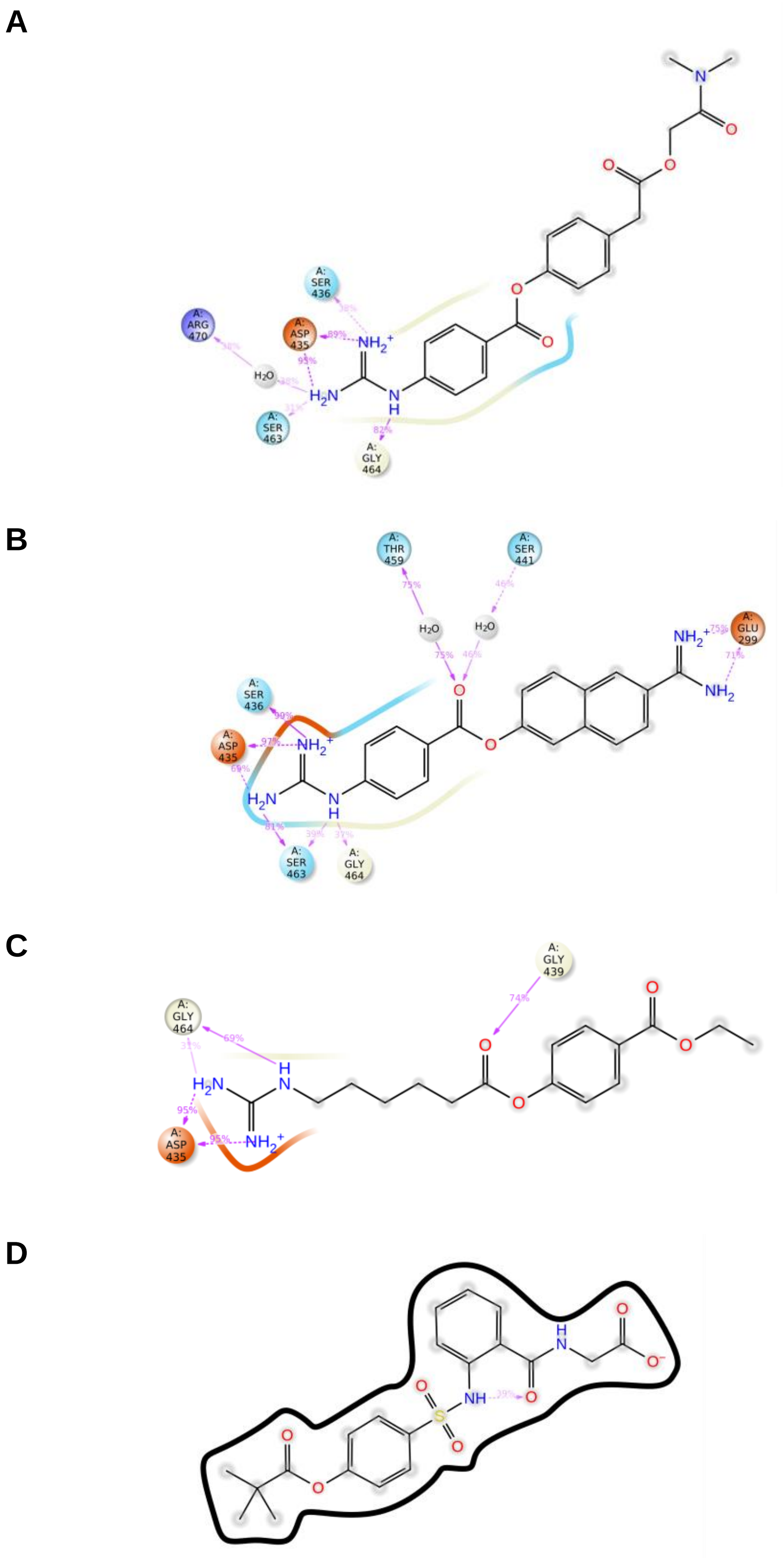

Figure S10. A schematic of detailed ligand atom interactions of (A) camostat, (B) nafamostat, (c) gabexate and (D) sivelestat with the protein residues of TMPS2 protein. Interactions that occur more than 30.0% of the simulation time in the selected trajectory (0 through 200 ns) are shown.

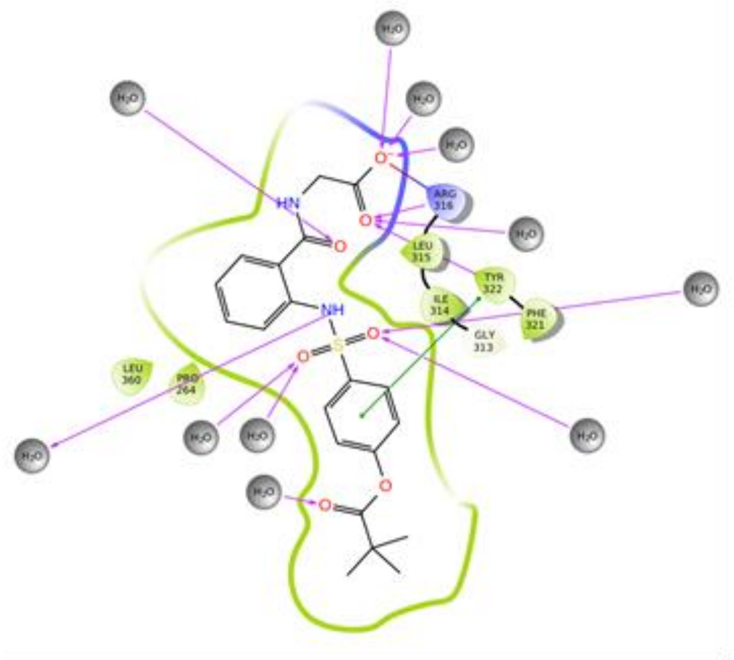

Figure S11. 2D Ligand interactions of sivelestat in TMPS2 protein.
